# The effect of stimulation interval on plasticity following repeated blocks of intermittent theta burst stimulation

**DOI:** 10.1038/s41598-018-26791-w

**Published:** 2018-06-04

**Authors:** Nga Yan Tse, Mitchell R. Goldsworthy, Michael C. Ridding, James P. Coxon, Paul B. Fitzgerald, Alex Fornito, Nigel C. Rogasch

**Affiliations:** 1 Brain and Mental Health Research Hub, School of Psychological Sciences, Monash Institute of Cognitive and Clinical Neuroscience, and Monash Biomedical Imaging, Monash University, Melbourne, Australia; 2 Neuromotor Plasticity and Development, Robinson Research Institute, Adelaide Medical School, The University of Adelaide, Adelaide, Australia; 3 School of Psychological Sciences, Monash Institute of Cognitive and Clinical Neuroscience, Monash University, Melbourne, Australia; 4 Epworth Healthcare, The Epworth Clinic and Monash Alfred Psychiatry Research Centre, Central Clinical School, Monash University, Melbourne, Australia

## Abstract

This study assessed the effect of interval duration on the direction and magnitude of changes in cortical excitability and inhibition when applying repeated blocks of intermittent theta burst stimulation (iTBS) over motor cortex. 15 participants received three different iTBS conditions on separate days: single iTBS; repeated iTBS with a 5 minute interval (iTBS-5-iTBS); and with a 15 minute interval (iTBS-15-iTBS). Changes in cortical excitability and short-interval cortical inhibition (SICI) were assessed via motor-evoked potentials (MEPs) before and up to 60 mins following stimulation. iTBS-15-iTBS increased MEP amplitude for up to 60 mins post stimulation, whereas iTBS-5-iTBS decreased MEP amplitude. In contrast, MEP amplitude was not altered by single iTBS. Despite the group level findings, only 53% of individuals showed facilitated MEPs following iTBS-15-iTBS, and only 40% inhibited MEPs following iTBS-5-iTBS. Modulation of SICI did not differ between conditions. These results suggest interval duration between spaced iTBS plays an important role in determining the direction of plasticity on excitatory, but not inhibitory circuits in human motor cortex. While repeated iTBS can increase the magnitude of MEP facilitation/inhibition in some individuals compared to single iTBS, the response to repeated iTBS appears variable between individuals in this small sample.

## Introduction

The capacity of the brain to modulate the strength of synaptic connections, commonly called synaptic plasticity, is a fundamental mechanism for healthy brain functioning, representing a key neural substrate for learning, memory, and development^1^. Changes in synaptic strength are governed by a variety of mechanisms, of which long-term potentiation (LTP) and depression (LTD) regulated by voltage-dependent n-methyl-d-aspartate (NMDA) receptors are the best characterised^2^. In *in vitro* studies of neural tissue, LTP is observed naturally following learning^3^, but can also be experimentally induced by external stimulation delivered at certain patterns mimicking natural brain rhythms. For example, theta-burst stimulation (TBS), which is a widely used plasticity-inducing paradigm, involves nesting high frequency (e.g. 100 Hz) stimulation pulses within slower frequencies (e.g. 5 Hz)^4,5^. However, the direction, magnitude, and duration of plasticity induced either naturally or experimentally is highly dependent on the history of synaptic activity, a phenomenon known as metaplasticity^6^. For instance, if a TBS paradigm which results in LTP is immediately followed by a second round of TBS, further LTP induction is blocked or even reversed^7,8^. Such interactions represent a homeostatic process that are thought to regulate the induction of LTP, preventing run-away changes in cortical excitability which could lead to excitotoxicity^6^. However, if the interval between the TBS protocols is increased, a longer more stable form of LTP termed “late-LTP” is induced^7^. This non-homeostatic form of metaplasticity is likely important for the persistent changes in synaptic strength required for learning and memory storage^9^. Thus, the timing of the interval between stimulation trains plays a critical role in determining the type of plasticity that can be induced^7,10^.

Plasticity can be studied in humans using non-invasive brain stimulation methods, such as transcranial magnetic stimulation (TMS)^11^. TBS protocols delivered with TMS (typically 50 Hz bursts at 5 Hz for 600 pulses) can alter cortical excitability beyond the period of stimulation^12^ and are blocked by NMDA antagonists^13^, reminiscent of LTP/LTD-like plasticity observed in animal experiments^14^. Early reports suggested that an intermittent pattern of stimulation (2 s on, 8 s off; iTBS) resulted in increased cortical excitability similar to LTP, whereas a continuous pattern (no off period; cTBS) resulted in decreased excitability similar to LTD^12^, findings which have been widely replicated (for reviews see^15,16^). However more recent studies have reported considerable inter-individual variation in response direction, with only ~50% of individuals responding in the expected direction to either protocol in sample sizes larger than 50 individuals^17–20^. When blocks of TBS are repeated in humans, evidence for both homeostatic (e.g. plasticity blocked or direction reversed), and non-homeostatic (e.g. increased magnitude) interactions have been reported (see Müller-Dahlhaus and Ziemann for review^21^), however the factors which govern the direction and magnitude of metaplasticity in humans are unclear. For instance, repeated blocks of cTBS with a 10 minute interval increase the magnitude and duration of MEP amplitude reduction compared with a single block of cTBS (i.e. non-homeostatic plasticity)^22^, reduce inter-individual variability in response direction^22^, and result in plasticity that is more robust against external influences^23^. However, the duration of the interval between repeated blocks of cTBS appears critical for determining the resultant direction of plasticity. For instance, an interval of 20 mins resulted in similar MEP reductions to a single block of cTBS^24^, whereas intervals of 2 or 5 mins resulted in no MEP change from baseline (i.e. blocked plasticity)^24^, and 15 mins a reversal in MEP change towards facilitation (i.e. homeostatic plasticity)^25^.

The influence of interval duration on plasticity following repeated blocks of iTBS is even more unclear, with conflicting results reported between studies (see Table 1). For example, repeated iTBS with an interval of 15 mins has resulted in enhanced MEP facilitation (3 blocks) or a similar level of facilitation (2 blocks) compared with a single block of iTBS in one study^26^, and a reversal in direction towards MEP depression in another study^25^. Comparing different intervals, larger MEP facilitation compared to single iTBS has been reported with a 10 min interval^27^, similar levels of facilitation with 2 min^24^ and 30 min^28^ intervals and no change/MEP inhibition with 5 and 20 min^24^ intervals compared to a single block of iTBS. Given the somewhat discordant findings, surprisingly only one study has systematically assessed the impact of altering the interval between repeated iTBS blocks within the same individuals^24^, and only one study has reported on inter-individual variability in response direction following repeated iTBS protocols^29^. Further, nearly all studies have focused on excitability, with only one study assessing whether inhibitory circuits^25^, which are also likely targeted by TBS^30^, show metaplasticity following repeated iTBS.

**Table 1:** Studies assessing repeated iTBS.

	iTBS protocol	Intervals	Measures	Findings
Gamboa^24^ (n = 10)	• 80% AMT, 600 pulses	• Single • 2 mins; • 5 mins; • 20 mins; 2 blocks	• MEPs (1 mV; mono, PA)	↑ MEPs (single) ↑ MEPs (2 mins) ↔ MEPs (5 mins) ↔ MEPs (20 mins) ↔ MEPs (2 mins)* ↓ MEPs (5 mins)* ↓ MEPs (20 mins)*
Murakami^25^ (n = 9, 80% AMT; n = 8, 70% AMT)	• 80% AMT (both blocks), 600 pulses • 70% AMT (first block) 80% AMT (second block)	• Single • 15 mins; 2 blocks	• MEPs (1 mV, 90–140% S1mV; mono, PA) • SICI (2 ms ISI; CS = 70–100% AMT; TS = S1mV)	↑ MEPs (single; 80% AMT)* ↓ MEPs (15 mins; 80% AMT)* ↔ SICI (single; 80% AMT)* ↓ SICI (15 mins; 80% AMT)* ↑ MEPs (single; 80% AMT)* ↔ MEPs (15 mins; 70% AMT)* ↔ SICI (single; 70% AMT)* ↔ SICI (15 mins; 70% AMT)*
Mastroeni^28^ (n = 29)	• 80% AMT, 600 pulses	• Single • 30 mins; 2 blocks	• MEPs (0.5 mV; mono, PA; bi AP-PA)	↑ MEPs mono (single) ↑ MEPs bi (single) ↑ MEPs mono (30 mins) ↑ MEPs bi (30 mins) ↔ MEPs mono (30 mins)* ↔ MEPs bi (30 mins)*
Nettekoven^26,29^ (n = 16)	• 70% RMT, 600 pulses	• Single, • 15 mins; 3 blocks	• MEPs (90–150% RMT; mono, PA)	↑ MEPs (single) ↑ MEPs (15 mins; 2 blocks) ↑ MEPs (15 mins; 3 blocks) ↔ MEPs (15 mins; 2 blocks)* ↑ MEPs (15 mins; 3 blocks)*
Opie^27^ (n = 16)	• 70% RMT, 600 pulses	• Single • 10 mins; 2 blocks	• MEPs (1 mV; mono, PA)	↔ MEPs (single) ↑ MEPs (10 mins) ↑ MEPs (10 mins)*

NB: Findings refer to changes from baseline in healthy, young adults. *Denotes post MEPs compared with primed MEPs as opposed to baseline. AMT, active motor threshold; bi, biphasic; CS, conditioning stimulus; MEP, motor evoked potential; mono, monophasic; RMT, resting motor threshold; S1mV, stimulus intensity giving 1 mV MEP; SICI, short-interval cortical inhibition; TS, test stimulus.

The aims of this study were twofold. First, we assessed whether the interval between consecutive repeated iTBS blocks affects the direction and magnitude of changes in cortical excitability following stimulation within the same individuals. We also assessed how variable changes in excitability were between individuals following different iTBS conditions. Second, we assessed whether plasticity on the inhibitory circuits targeted by iTBS is also altered following repeated iTBS at different intervals. Given the findings from animal studies^7^, we hypothesised that shorter intervals (5 mins) would lead to reductions in MEP amplitude and SICI, whereas longer intervals (15 mins) would result in increased MEP and SICI facilitation compared to a single block of iTBS.

## Results

### Overview

**Figure 1:**
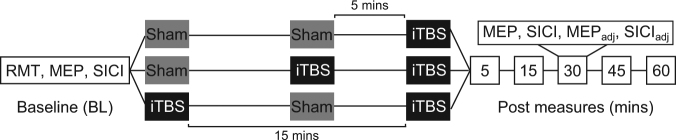
Diagram demonstrating the study protocol. Participants received three different iTBS conditions on separate days: single iTBS (top); iTBS-5-iTBS (middle); and iTBS-15-iTBS (bottom). Note that time is not drawn to scale for baseline and post measures.

15 healthy participants completed the study. To address the primary and secondary aim of the study, changes in cortical excitability and inhibition were assessed for up to 60 mins after three different iTBS conditions applied to the left motor cortex: a single iTBS session (iTBS); iTBS primed by another iTBS session 5 minutes prior (iTBS-5-iTBS); and iTBS primed by another iTBS session 15 minutes prior (iTBS-15-iTBS). Cortical excitability was indexed as the peak-to-peak amplitude of motor-evoked potentials (MEPs) recorded from the first dorsal interosseous muscle with stimulator intensity set to give an MEP of ~1 mV in amplitude (S1mV) at baseline. Cortical inhibition was indexed using the short interval cortical inhibition paradigm (SICI) with an interstimulus interval of 2 ms, the conditioning intensity set to 70% of resting motor threshold (RMT) and the test intensity set to S1mV. To account for possible effects of altered MEP amplitude on SICI following iTBS, the MEP amplitude was adjusted to give an amplitude of ~1 mV (MEP_adj_) and SICI was re-assessed using this adjusted test intensity (SICI_adj_). To ensure each condition was as similar as possible, sham iTBS was applied at the 5 and 15 min intervals in the conditions not requiring active stimulation at these time points (Fig. 1).

### Baseline measures

There were no differences in RMT (F_2,14_ = 0.3, p = 0.736), S1mV intensity (F_2,14_ = 0.3, p = 0.764), MEP amplitude (F_2,14_ = 1.9, p = 0.174), or SICI (F_2,14_ = 1.4, p = 0.246) between conditions at baseline.

### Cortical excitation following single and spaced iTBS

**Figure 2:**
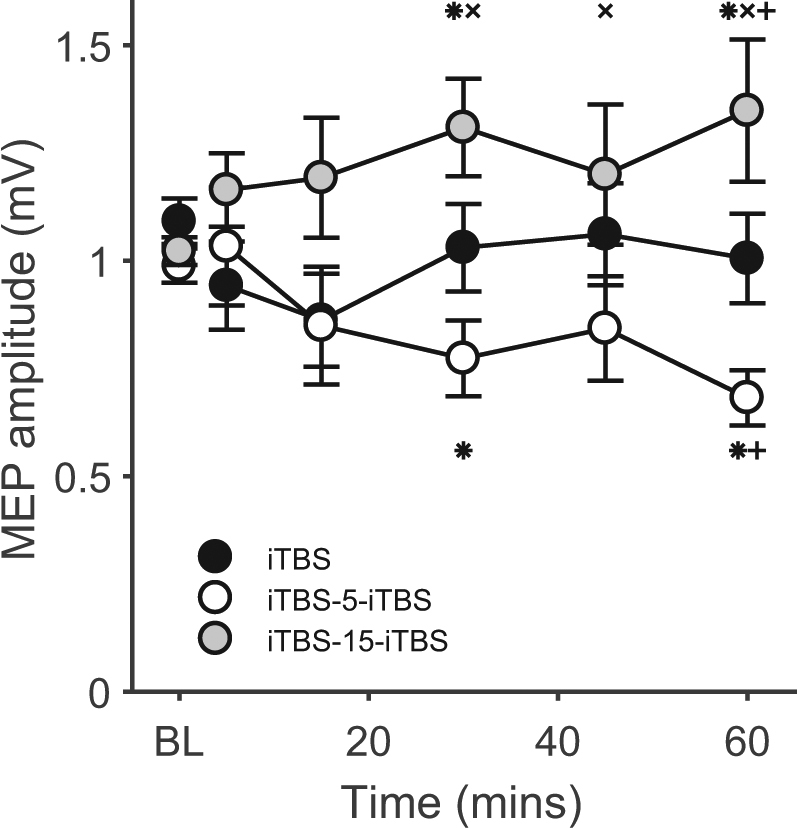
MEP amplitudes following single and spaced iTBS. BL = baseline. *p < 0.05 compared to BL; ^×^p < 0.05 compared to iTBS-5-iTBS; ^+^p < 0.05 compared to single iTBS.

To assess whether the interval between repeated blocks of iTBS influences the direction of plasticity, we first compared iTBS-induced changes in MEP amplitude between conditions. RMANOVA on MEP amplitude revealed a significant main effect of CONDITION (F_2,14_ = 7.4, p = 0.003, η^2^ = 0.35), no effect of TIME (F_5,14_ = 0.3, p = 0.933, η^2^ = 0.02), but most importantly, a significant CONDITION × TIME interaction (F_10,14_ = 2.4, p = 0.011, η^2^ = 0.15). For the main effect of CONDITION, post-hoc tests showed that MEP amplitudes were larger following iTBS-15-iTBS than both single iTBS (p = 0.042) and iTBS-5-iTBS (p = 0.004). For the interaction, post-hoc tests showed that MEP amplitudes were larger following iTBS-15-iTBS compared to iTBS-5-iTBS at 30 mins (p = 0.001), 45 mins (p = 0.045) and 60 mins (p = 0.002), and were larger following iTBS-15-iTBS (p = 0.034) and smaller following iTBS-5-iTBS (p = 0.012) compared to single iTBS at 60 mins (Fig. 2). Secondary analyses confirmed that, compared to baseline, MEP amplitudes were increased at 30 mins (p = 0.033) and 60 mins (p = 0.049) following iTBS-15-iTBS, and decreased at 30 mins (p = 0.030) and 60 mins (p < 0.001) following iTBS-5-iTBS. No changes were identified following single iTBS (all p > 0.05). These results suggest that the spacing interval between repeated applications of iTBS effects the direction of excitability change, particularly at later time points.

### Individual response to spaced iTBS

**Figure 3:**
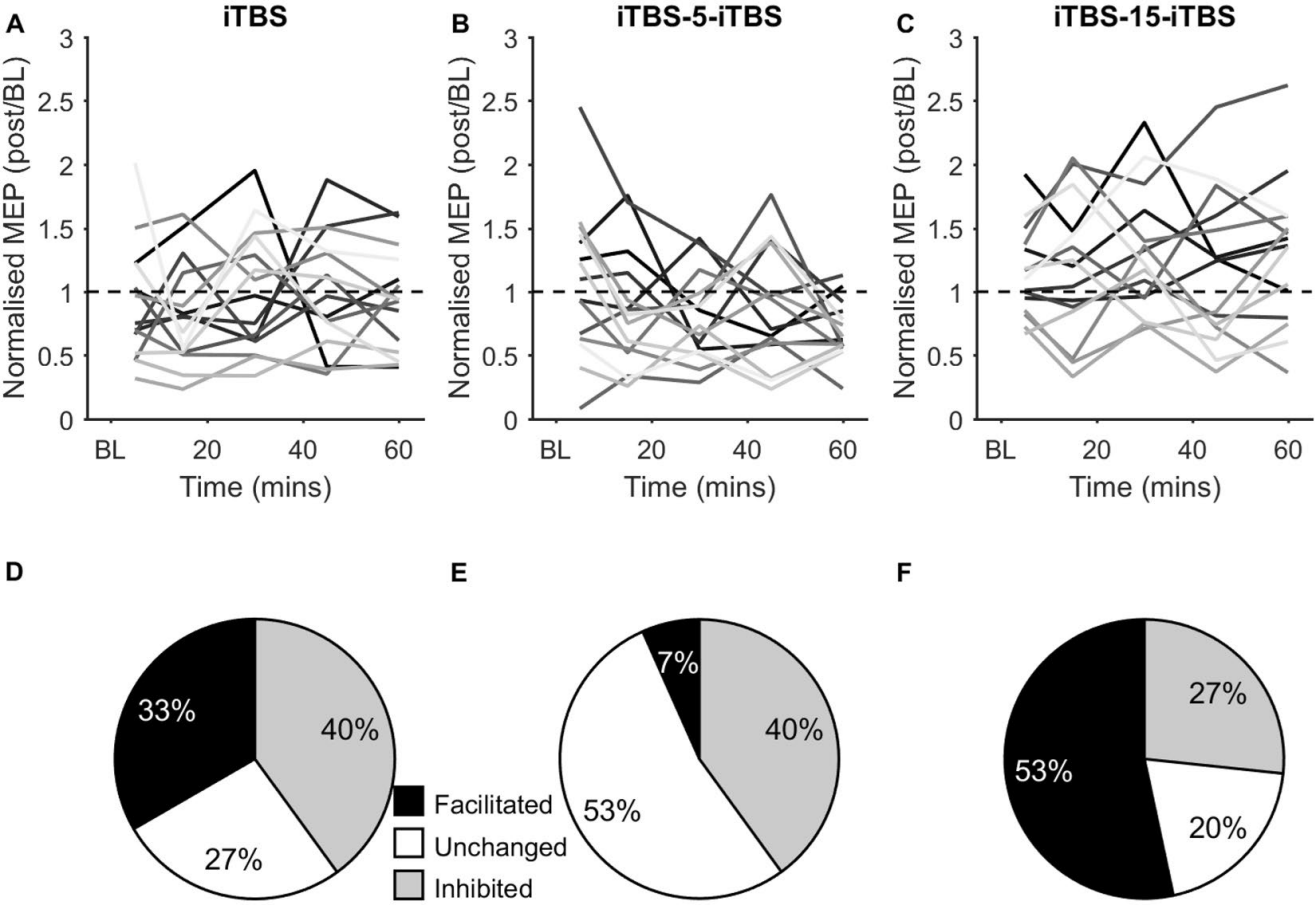
Individual responses to single and spaced iTBS. (**A**–**C**) Normalised changes in MEP amplitude following different iTBS conditions in individuals, represented as different shades of grey. Changes in MEP amplitude have been normalised to baseline values, with score >1 indicating an increase, and <1 a decrease in excitability. (**D**–**F**) Percentage of individuals showing facilitated, unchanged, or inhibited MEPs following each iTBS condition.

Figure 3 shows individual responses to iTBS for each condition normalised to baseline. Variability between individuals is apparent in response to both single and spaced iTBS conditions (Fig. 3A–C). To quantify the percentage of individuals in which MEPs were facilitated, inhibited, or unchanged following each condition, normalised changes in MEP amplitude were averaged across post iTBS time points to generate a grand average value for each individual. Individuals with a grand average value >1.1 were considered to have facilitated MEPs, <0.9 inhibited MEPs, and 0.9 < grand average < 1.1 unchanged MEPs following each iTBS condition according to a previous definition^20,29^. Facilitated MEPs were observed in 33% of individuals following single iTBS, whereas 40% showed inhibited MEPs (Fig. 3D). Although iTBS-15-iTBS resulted in a larger increase in MEP amplitude at the group level, the number of facilitators at the individual level was only marginally higher than single iTBS, with facilitated MEPs observed in 53% of individuals and inhibited MEPs in 27% (Fig. 3F). Similar to single iTBS, inhibited MEPs were observed in 40% of individuals following iTBS-5-iTBS, however only 7% showed facilitated MEPs (Fig. 3E).

### Correlations between changes in MEP amplitude following spaced iTBS

**Figure 4:**
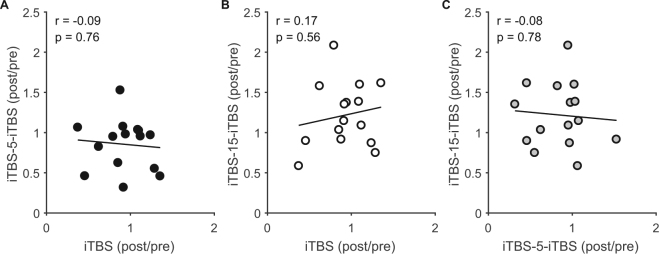
Relationship between single and spaced iTBS. Correlations between the normalised grand average MEP amplitude following different iTBS conditions.

To assess whether response profiles were similar between different iTBS conditions, we correlated the grand average response between single and spaced iTBS. There was no relationship between the responses to different conditions for any pairing (iTBS v iTBS-5-iTBS, p = 0.76; iTBS v iTBS-15-iTBS, p = 0.56; iTBS-5-iTBS-iTBS v iTBS-15-iTBS, p = 0.78; Fig. 4), suggesting that different mechanisms may underlie the variability in response to single and spaced iTBS.

### Cortical inhibition following single and spaced iTBS

**Figure 5:**
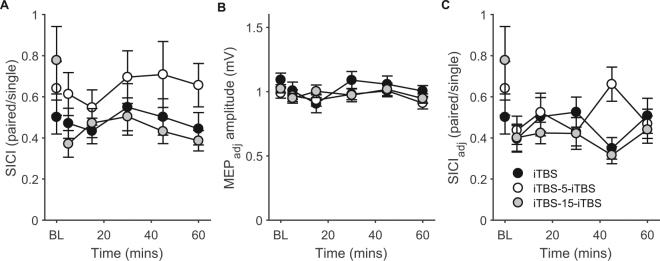
SICI following single and spaced iTBS. (**A**) Changes in SICI following iTBS without adjusting test MEP amplitude. (**B**) MEP amplitudes at each time point after adjusting TMS intensity to give a 1 mv response (MEP_adj_). (**C**) Changes in SICI following iTBS after adjusting test MEP amplitude (SICI_adj_).

To assess whether similar interactions between plasticity and interval duration occur within inhibitory populations, we also assessed changes in SICI following single and spaced iTBS. RMANOVA on SICI revealed no main effect of CONDITION (F_2,14_ = 3.3, p = 0.052, η^2^ = 0.19) or TIME (F_5,14_ = 1.5, p = 0.206, η^2^ = 0.10), and no CONDITION × TIME interaction (F_10,14_ = 1.3, p = 0.274, η^2^ = 0.09) (Fig. 5A). To control for possible confounding effects of iTBS-induced changes in MEP amplitude on SICI, we repeated SICI measurements (SICI_adj_) with MEP amplitude adjusted to 1 mV (MEP_adj_). This adjustment to the test stimulus intensity successfully matched the non-conditioned response amplitude as evidenced by there being no main effect of CONDITION (F_2,14_ = 1.9, p = 0.164, η^2^ = 0.12) and no CONDITION × TIME interaction (F_10,14_ = 0.4, p = 0.944, η^2^ = 0.02) on adjusted MEP amplitude following iTBS (Fig. 5B). As with unadjusted SICI, there was no main effect of CONDITION (F_2,14_ = 1.2, p = 0.238, η^2^ = 0.09), or CONDITION × TIME interaction (F_10,14_ = 3.3, p = 0.274, η^2^ = 0.09) on SICI_adj_, however there was a main effect of TIME (F_5,14_ = 1.5, p = 0.042, η^2^ = 0.19). Averaged across conditions, SICI_adj_ increased at 5 mins (p = 0.009), 30 mins (p = 0.034), 45 mins (p = 0.019), and 60 mins (p = 0.004) following iTBS (Fig. 5C). These findings suggest that inhibitory circuits do not show similar changes in plasticity following spaced iTBS at different intervals as excitatory populations.

Although there was no significant difference in baseline SICI between conditions, inspection of Fig. 5 suggested that baseline SICI was qualitatively lower (i.e. closer to 1) in the iTBS-15-iTBS condition compared to single iTBS. To ensure that lower SICI was not driving the increase in MEP amplitude in the iTBS-15-iTBS condition, we correlated: (1) SICI strength at baseline with grand average change in normalised MEP amplitude following iTBS; and (2) differences in baseline SICI strength between the single and iTBS-15-iTBS condition with differences in normalised MEP amplitude change between conditions. There was no significant correlation between baseline SICI and normalised changes in MEP amplitude for combined single and iTBS-15-iTBS conditions (r = 0.12, p = 0.54) or iTBS-15-iTBS alone (r = 0.07, p = 0.81). Furthermore, there was no significant correlation between the difference in baseline SICI and the difference in normalised MEP change when comparing the single and iTBS-15-iTBS condition (r = 0.09, p = 0.74), suggesting between session differences in baseline SICI were not driving the larger increases in MEP amplitude following iTBS-15-iTBS.

## Discussion

Repeating blocks of TBS can influence the magnitude and direction of plasticity in humans, however the factors determining the nature of this interaction are not well defined. Our findings add to the somewhat confusing literature surrounding repeated blocks of iTBS by assessing the influence of interval duration on changes in both cortical excitability and inhibition within the same individuals, as well as characterising the individual variability in response to repeated iTBS at multiple intervals. In this study, we have found that the interval duration between repeated blocks of iTBS impacts the resulting plasticity direction, with shorter intervals (5 mins) reducing cortical excitability, and longer intervals (15 mins) increasing excitability at the group level. Unlike cortical excitability, we could not find any evidence that plasticity of inhibitory circuits is altered following repeated blocks of iTBS. Despite changes in MEP amplitude at the group level following repeated iTBS compared to single iTBS, MEPs were altered in only approximately half of the sample. Although the sample size is small in the current study, this preliminary evidence suggests variability in response direction to both single and repeated iTBS between individuals.

A single block of iTBS (600 pulses) is reported to increase cortical excitability indexed by MEP amplitude for ~30 mins following stimulation^12^, changes which are blocked by NMDA receptor antagonists^13^. As such, cortical excitability increases following iTBS are thought to reflect LTP-like plasticity mechanisms^15,16^. We could not find any evidence for changes in cortical excitability following a single block of iTBS at the group level. Indeed, only 33% of individuals in our study showed facilitated MEPs (e.g. MEP amplitude change > 1.1 compared to baseline), with 40% responding in the opposite direction. This finding may seem contradictory to the expected facilitatory effect of iTBS^12^, and could reflect methodological differences between our iTBS protocol and the original^12^, including the stimulation intensity (70% RMT vs 80% AMT), baseline muscle activity (relaxed vs contraction), and the pulse shape used to assess changes in MEPs (biphasic vs monophasic). However, several recent studies with large sample sizes (N > 50) using the original iTBS protocol have reported considerable inter-individual variability in response to iTBS, resulting in no net differences in excitability at the group level^17,18^. A recent meta-analysis of 59 iTBS studies found that, while there was evidence for MEP facilitation following iTBS, the effect size of MEP facilitation reduced with increasing sample sizes^31^. This finding provides strong evidence for publication bias in the iTBS literature, and suggests inter-individual variability in response to a single block of iTBS may be larger than initially expected. Indeed, a recent position paper on plasticity following non-invasive brain stimulation techniques conceded that the expected ‘response rate’ may be less than 50% for most brain stimulation methods including iTBS^16^. The reasons for the variability in response to single iTBS are likely multi-faceted and could include both methodological (e.g. intensity of TBS used, brain-state at time of TBS) and/or biological (e.g. genetic variability, age) factors^15,16^. Although only in a small sample, our findings are consistent with a growing body of literature suggesting large inter-individual variability in response to a single block of iTBS using either the original protocol, or variants on this protocol.

In animal slice studies, the instability/variability of plasticity induced by a single block of stimulation is overcome by administering multiple blocks of stimulation to induce late-LTP^10^, in which the interval duration plays an important role in determining the magnitude and direction of plasticity^7^. In humans, two blocks of cTBS at 10 minute intervals results in a more reliable and longer lasting reduction in MEP amplitude^22^ compared with a single block alone. Importantly, the plasticity following spaced cTBS at 10 mins is resistant to reversal by subsequent physiological activity, such as muscle contractions, suggesting consolidation of LTD-like plasticity^23^. In contrast, intervals of 20 mins resulted in a reduction of MEP amplitude similar in magnitude to a single block of cTBS^24^, whereas interval durations of 2^24^, 5^24^, 15^25^ or 30^28^ mins resulted in no change/MEP facilitation following spaced cTBS, consistent with homeostatic plasticity.

The interactions between repeated blocks of iTBS on plasticity is also unclear. Previous studies have reported: (1) MEP facilitation which is larger in magnitude compared to single iTBS following repeated iTBS with a 10 minute interval^27^, (2) MEP facilitation comparable to single iTBS with intervals of 2^24^ or 30 mins^28^, and (3) no change/MEP inhibition with intervals of 5 or 20 mins^24^. Our finding of a reduction in MEP amplitude following repeated iTBS with an interval of 5 mins is therefore broadly consistent with the only other previous study using this interval. Repeated iTBS with an interval of 15 mins has resulted in inconsistent findings, with MEP inhibition reported after two blocks of stimulation in one study^25^, and a similar level of MEP facilitation to single iTBS after two blocks, and larger MEP facilitation after three blocks of stimulation in another^26^. Furthermore, a study measuring changes in TMS-evoked cortical potentials using electroencephalography following prefrontal single and repeated iTBS spaced 15 mins apart reported a similar increases in amplitude between the conditions^32^. Our finding that two blocks of repeated iTBS with a 15 mins interval resulted in a larger MEP facilitation compared to single iTBS at the group level after 30 mins post stimulation, is thus consistent with one previous studies, but not another. Taken together, our findings suggest that the interval between repeated blocks of iTBS is important for determining the direction of subsequent plasticity, however the response direction can be variable.

A challenge when comparing the impact of interval duration following repeated iTBS protocols between previous studies is the differences in iTBS methods employed. For example, studies have used different stimulation intensities (e.g. 80% AMT, 70% RMT), which involve different levels of prior muscle activity (e.g. active vs relaxed), and different outcome measures to quantify plasticity (e.g. MEPs at different intensities, monophasic or biphasic pulse shapes; see Table 1 for overivew). Performing muscle contractions prior to single TBS (e.g. to obtain AMT) can influence subsequent plasticity induction^22,33–35^, therefore complicating the interpretation of changes in excitability. Alternatively, 70% RMT typically results in a stimulation intensity slightly higher than 80% AMT on average^22^, which can impact the direction of subsequent plasticity interactions. For instance, Doeltgen and Ridding (2011) found that excitability increased following a 300 pulse cTBS paradigm at 70% RMT, but decreased with 65% RMT cTBS (approximately equivalent to 80% AMT)^36^. The impact of these methodological decisions on plasticity outcomes following repeated TBS protocols remains largely unexplored, especially for iTBS. Goldsworthy and colleagues showed that using 80% AMT to set stimulation intensity abolished the MEP amplitude reductions observed following repeated cTBS at 10 minute intervals compared with either 70% or 65% RMT, suggesting that prior muscle activity could influence repeated cTBS outcomes^22^. Although it is unclear whether the same is true for iTBS, we designed our study to avoid the voluntary muscle activity associated with determination of AMT. As such, a systematic comparison of the impact of different parameters, such as stimulation threshold methods, on plasticity outcomes following repeated iTBS is required.

It is unclear from the current study what mechanisms underlie the switch in plasticity direction following repeated blocks of iTBS with different interval durations. In rat hippocampus, priming stimulation can inhibit or reverse the induction of subsequent LTP^8,37^, and facilitate LTD^38^ across individual synapses. Importantly, priming stimulation does not need to induce LTP for these homeostatic metaplastic interactions to occur^8,37^. However, this LTP blocking/reversal can be overcome by introducing a gap between stimulation trains^7,8,10,39^, resulting in a longer, more stable form of LTP deemed late-LTP. Although speculative, our results could reflect this critical temporal window which separates homeostatic from non-homeostatic metaplasticity. An important caveat when comparing animal to human plasticity studies is the differences in scale, with animal work typically describing plasticity induced at individual synapses, whereas human work involves plasticity across large neural populations following TMS. Rodent models using TMS have demonstrated that the concentration of vesicular glutamate transporters increase with repeated blocks of iTBS spaced by 15 mins, indicative of increased cortical excitability as seen in our study^40^. Such animal models using TMS will be important to uncover the precise mechanisms responsible for the effect of interval duration on the outcomes of iTBS.

Another motivation for exploring repeated iTBS is to assess whether this method reduces inter-individual variability compared to single iTBS. Indeed, one study found that repeated cTBS resulted in 100% of participants showing reduced MEP amplitude compared to 58% with single cTBS^22^. We found that 53% of participants had a facilitatory response following iTBS-15-iTBS, while 27% showed an inhibitory response. For iTBS-5-iTBS, 40% of individuals showed an inhibitory response, while MEP amplitude remained unchanged in 53% of individuals. While the response to iTBS-15-iTBS was a marginal increase on the percentage of individuals with facilitated MEPs following single iTBS (33%), these findings suggest the response to repeated iTBS is variable. A similar result was reported following three blocks of iTBS separated by 15 mins, which showed that, while repeated iTBS increased the magnitude of the response at the group level, it did not convert those that responded with inhibited MEPs to facilitation^29^. Although the sample size is small in this study, our results provide preliminary evidence that the response to repeated iTBS is variable at the individual level. This finding requires replication in a larger sample.

In addition to altering cortical excitability, there is also some evidence that iTBS can alter inhibitory circuits. Huang and colleagues reported that a single block of iTBS increased GABA_A_-mediated neurotransmission as assessed using SICI^12^, however, a recent meta-analysis found no evidence for changes in SICI following iTBS across 13 datasets^31^. We also found no evidence for changes in SICI following either single or spaced iTBS. However we did find a general increase in SICI when we adjusted the test MEP amplitude to account for iTBS-induced changes in cortical excitability, which did not differ between conditions. Goldsworthy and colleagues also found that single and spaced cTBS had similar effects on inhibitory circuits, with both conditions decreasing SICI^41^. In contrast, Murakami and colleagues found that spaced iTBS with a 15 minute interval resulted in reduced SICI compared to a single block of iTBS^25^. The reasons for this discrepancy are unclear, however differences in voluntary contractions prior to TBS or differences in the absolute stimulation intensity may have influenced the outcomes between studies. We also assessed whether baseline SICI could explain the facilitation in MEPs following iTBS-15-iTBS, as lower inhibition can influence subsequent plasticity. In line with other studies using single iTBS^18^, baseline SICI did not predict response to iTBS-15-iTBS. Taken together, our results suggest that spaced iTBS does not result in metaplasticity of inhibitory circuits, at least at the intervals tested.

There are several limitations to the current study. First, we did not assess changes in MEP amplitude or SICI directly following the priming block of stimulation, as we did not want to inadvertently disrupt any ongoing plasticity. Although results from the single iTBS condition suggest that MEP amplitude and SICI were unaffected by one block of stimulation in our sample, we are unable to definitely assess whether priming stimulation did or did not alter cortical excitability or inhibition. Importantly, metaplasticity is not dependent on changes in synaptic efficacy following priming^6^, and several studies in human motor cortex have reported metaplasticity following repeated TBS blocks without excitability changes following the priming block^22,25,27^. Second, we used biphasic TMS pulses to assess MEP amplitude and SICI following iTBS, whereas the majority of other studies have used monophasic pulses for these assessments (see Table 1). The choice to use biphasic pulses was deliberate, as we wanted to more accurately assess the neural populations targeted by iTBS, which are stimulated using biphasic pulses. Changes in MEP amplitude following TBS assessed with biphasic pulses (AP-PA current flow in cortex) are highly comparable to those using monophasic pulses (PA current flow in cortex)^28,42^. As such, it is unlikely this choice had a major impact on the study outcomes and conclusions. Third, we only assessed cortical excitability/inhibition for 60 mins following iTBS, at which point MEP amplitude had not returned to baseline levels. It is therefore unclear how long repeated blocks of iTBS either increased/decreased cortical excitability, which is of interest for future studies.

## Conclusions

We provide evidence that the interval duration between repeated blocks of iTBS over human motor cortex can impact the direction of plasticity on excitatory circuits, with shorter intervals at 5 mins favouring MEP inhibition, and longer intervals at 15 mins MEP facilitation. In contrast, repeated blocks of iTBS did not alter plasticity of inhibitory circuits. Although repeated iTBS altered the magnitude of MEP changes at the group level compared to single iTBS, only approximately half of the sample showed consistent changes in MEP amplitude relative to baseline, providing preliminary evidence for inter-individual variability in response to repeated iTBS. Understanding the determinants of this variability in response to iTBS will be essential for designing stimulation paradigms to more consistently drive plasticity in a given individual.

## Methods

### Participants

20 healthy participants were recruited from the wider community. Prior to the experiment, all participants were screened for any contraindications to TMS based on the TMS safety guidelines^43^, and provided written informed consent. Five participants were withdrawn from the study due to high stimulation thresholds (n = 2), inability to complete all sessions (n = 1), light-headedness following resting motor threshold (n = 1), and persistent muscle activity during TBS (n = 1). As such, 15 participants completed all three experimental conditions (mean age = 24.8 ± 4 years; 7 females; 1 left handed). The study was approved by the Monash University Human Research Ethics Committee, and conducted in accordance with the Declaration of Helsinki.

### Electromyography

Participants were seated comfortably in a chair with their right hand and arm resting on a pillow. Surface electromyography (EMG) was recorded from the right first dorsal interosseous (FDI) muscle. Ag-AgCl electrode pairs were placed in a belly-tendon montage over the FDI muscle, with a ground electrode placed over the distal surface of the hand. EMG signals were recorded from 100 ms before to 500 ms after each TMS pulse. Signals were amplified (×1000), bandpass filtered (10–1000 Hz), and digitized (5 kHz), and stored on a computer for offline analysis (Powerlab 26 T, ADInstruments Ltd., New Zealand).

### Transcranial magnetic stimulation

TMS was applied through a figure-of-eight coil (C-B60; 75 mm outer diameter) connected to a MagPro X100 stimulator (MagVenture, Denmark). The current waveform was biphasic for all conditions. Biphasic pulses were chosen over monophasic pulses to more accurately index cortical excitability from the neural pool stimulated using TBS (which uses biphasic pulses). The coil was held tangentially to the skull with the handle pointing backwards, at an angle of 45° to the sagittal plane, such that an anterior-posterior followed by a posterior-anterior current flow (AP-PA) was induced in the underlying cortex. The coil was held over the hand area of the left motor cortex. The scalp position resulting in the most consistent and largest MEP in the FDI muscle (i.e. the motor ‘hotspot’) was determined and used throughout the session. This position was marked on the participant’s scalp with a water-soluble pen in order to keep coil positioning consistent. The resting motor threshold (RMT) was then determined as the minimum intensity necessary to elicit at least 5 out of 10 MEPs with a peak-to-peak amplitude greater than 50 µV while the target muscle is relaxed. The stimulus intensity was then increased to induce an MEP of ~1 mV in amplitude (stimulus intensity; S1mV). This stimulus intensity was used throughout the experiment to index changes in cortical excitability.

Intracortical inhibition was assessed using the paired-pulse short-interval cortical inhibition (SICI) paradigm. SICI was applied with an inter-stimulus interval of 2 ms. This interval was chosen to index true GABA_A_-mediated neurotransmission and limit contamination from short-interval cortical facilitation^44^. The conditioning pulse intensity was set at 70% of RMT, and the test pulse intensity at S1mV. The conditioning intensity was chosen to match the intensity used for iTBS (see below), thereby allowing a direct evaluation of excitability changes induced in the neural population targeted by TBS. As the amplitude of the test MEP can influence SICI magnitude^45^, SICI recordings were repeated in the post iTBS blocks with the test pulse stimulus adjusted to provide an MEP of ~1 mV in amplitude to a single TMS pulse (MEP_adj_ and SICI_adj_). MEPs and SICI were assessed at baseline, and at 5, 15, 30, 45, and 60 mins post the main iTBS session. 15 trials were collected for each measurement at a frequency of 0.2 Hz.

### Intermittent theta burst stimulation

iTBS was delivered over the FDI motor hotspot using an actively cooled figure-of-eight magnetic coil (MCF-B65, MagVenture; 75 mm outer diameter). RMT was re-measured using the cooled coil. Stimulation consisted of a burst of three pulses administered at 50 Hz, repeated at a frequency of 5 Hz, delivered in 2 s trains followed by an 8 s interval for a total of 600 pulses^12^. Stimulation intensity was set at 70% of RMT. The more common TBS intensity of 80% active motor threshold (AMT) was not used to avoid voluntary contractions prior to TBS, as several studies have suggested that such contractions can interfere with subsequent TBS-induced plasticity^33–35^.

Sham iTBS was achieved by rotating the coil head 90° around its axis so the magnetic field ran perpendicular to the scalp, and the coil wing rested over the motor hotspot. In between stimulation and recording blocks, participants were instructed to remain as relaxed as possible and to avoid moving their hands. The three iTBS conditions were randomised and completed on different days by the same experimenters separated by at least 72 hours.

### Data analysis

Trials with background muscle activity (root-mean-squared EMG > 10 μV in the 100 ms prior to stimulation) were excluded from further analysis. To index changes in cortical excitability following iTBS, peak-to-peak MEP amplitudes were averaged across trials in each recording block following single pulse TMS. To compare changes in cortical excitability between each condition, post MEP amplitudes were normalised to baseline, and averaged across post iTBS time period for each individual (grand average response). SICI was quantified by dividing the mean conditioned peak-to-peak MEP amplitude (e.g. the test MEP following paired-pulse TMS), by the mean peak-to-peak amplitude of the single pulse MEP alone. For the post iTBS blocks, this was repeated for trials using the adjusted test MEP amplitude to calculate SICI_adj_. Using this formula, values close to 0 indicate strong inhibition, whereas values close to 1 indicate weak inhibition.

### Statistics

One-way analysis of variance (ANOVA) was used to compare baseline RMT, S1mV, MEP amplitude and SICI between conditions. To examine the effect of different spaced iTBS intervals on cortical excitability and inhibition, 3 × 6 repeated measures ANOVAs (RMANOVAs) were used to test the main effects of CONDITION (iTBS, iTBS-5-iTBS, iTBS-15-iTBS) and TIME (baseline, 5, 15, 30, 45, 60) on MEP amplitude, SICI, MEP_adj_ amplitude and SICI_adj_. The Shapiro-Wilk test was used to assess normality and detected outliers in non-normal data were winsorised to the next highest value. Mauchly’s test was used to assess sphericity and the Greenhouse-Geiser correction applied if necessary. In the case of significant main effects or interactions, targeted post-hoc testing was applied using Fisher’s PLSD test. Pearson’s correlations were used to assess whether there was any relationship between grand average changes in MEP amplitude between conditions. In all tests, a value of p ≤ 0.05 was considered statistically significant. All data are expressed as mean ± SD in the text and mean ± SEM in the figures.

### Data availability

The datasets generated during and/or analysed during the current study are available from the corresponding author on reasonable request.

## References

[1] Citri A, Malenka RC (2008). Synaptic plasticity: multiple forms, functions, and mechanisms. Neuropsychopharmacology.

[2] Malenka RC, Bear MF (2004). LTP and LTD: an embarrassment of riches. Neuron.

[3] Rioult-Pedotti MS, Friedman D, Hess G, Donoghue JP (1998). Strengthening of horizontal cortical connections following skill learning. Nat. Neurosci..

[4] Larson J, Wong D, Lynch G (1986). Patterned stimulation at the theta frequency is optimal for the induction of hippocampal long-term potentiation. Brain Res..

[5] Kirkwood A, Dudek SM, Gold JT, Aizenman CD, Bear MF (1993). Common forms of synaptic plasticity in the hippocampus and neocortex *in vitro*. Science.

[6] Abraham WC (2008). Metaplasticity: tuning synapses and networks for plasticity. Nat. Rev. Neurosci..

[7] Zhou Q, Tao HW, Poo MM (2003). Reversal and Stabilization of Synaptic Modifications in a Developing Visual System. Science.

[8] Abraham WC, Huggett A (1997). Induction and reversal of long-term potentiation by repeated high-frequency stimulation in rat hippocampal slices. Hippocampus.

[9] Abel T (1997). Genetic demonstration of a role for PKA in the late phase of LTP and in hippocampus-based long-term memory. Cell.

[10] Abraham WC, Logan B, Greenwood JM, Dragunow M (2002). Induction and experience-dependent consolidation of stable long-term potentiation lasting months in the hippocampus. J. Neurosci..

[11] Hallett M (2007). Transcranial Magnetic Stimulation: A Primer. Neuron.

[12] Huang Y-Z, Edwards MJ, Rounis E, Bhatia KP, Rothwell JC (2005). Theta Burst Stimulation of the Human Motor Cortex. Neuron.

[13] Huang Y-Z, Chen R-S, Rothwell JC, Wen H-Y (2007). The after-effect of human theta burst stimulation is NMDA receptor dependent. Clin. Neurophysiol..

[14] Hoogendam JM, Ramakers GMJ, Di Lazzaro V (2010). Physiology of repetitive transcranial magnetic stimulation of the human brain. Brain Stimul..

[15] Suppa A (2016). Ten Years of Theta Burst Stimulation in Humans: Established Knowledge, Unknowns and Prospects. Brain Stimul..

[16] Huang Y-Z (2017). Plasticity induced by non-invasive transcranial brain stimulation: A position paper. Clin. Neurophysiol..

[17] Hamada M, Murase N, Hasan A, Balaratnam M, Rothwell JC (2013). The role of interneuron networks in driving human motor cortical plasticity. Cereb. Cortex.

[18] López-Alonso V, Cheeran B, Río-Rodríguez D, Fernández-Del-Olmo M (2014). Inter-individual variability in response to non-invasive brain stimulation paradigms. Brain Stimul..

[19] Vallence A-M (2015). Inter- and intra-subject variability of motor cortex plasticity following continuous theta-burst stimulation. Neuroscience.

[20] Hinder MR (2014). Inter- and Intra-individual variability following intermittent theta burst stimulation: implications for rehabilitation and recovery. Brain Stimul..

[21] Müller-Dahlhaus F, Ziemann U (2015). Metaplasticity in human cortex. Neuroscientist.

[22] Goldsworthy MR, Pitcher JB, Ridding MC (2012). The application of spaced theta burst protocols induces long-lasting neuroplastic changes in the human motor cortex. Eur. J. Neurosci..

[23] Goldsworthy MR, Müller-Dahlhaus F, Ridding MC, Ziemann U (2015). Resistant Against De-depression: LTD-Like Plasticity in the Human Motor Cortex Induced by Spaced cTBS. Cereb. Cortex.

[24] Gamboa OL (2011). Impact of repetitive theta burst stimulation on motor cortex excitability. Brain Stimul..

[25] Murakami T, Müller-Dahlhaus F, Lu M-K, Ziemann U (2012). Homeostatic metaplasticity of corticospinal excitatory and intracortical inhibitory neural circuits in human motor cortex. J. Physiol..

[26] Nettekoven C (2014). Dose-dependent effects of theta burst rTMS on cortical excitability and resting-state connectivity of the human motor system. J. Neurosci..

[27] Opie GM, Vosnakis E, Ridding MC, Ziemann U, Semmler JG (2017). Priming theta burst stimulation enhances motor cortex plasticity in young but not old adults. Brain Stimul..

[28] Mastroeni C (2013). Brain-Derived Neurotrophic Factor – A Major Player in Stimulation-Induced Homeostatic Metaplasticity of Human Motor Cortex?. PLoS One.

[29] Nettekoven C (2015). Inter-individual variability in cortical excitability and motor network connectivity following multiple blocks of rTMS. Neuroimage.

[30] McAllister SM, Rothwell JC, Ridding MC (2009). Selective modulation of intracortical inhibition by low-intensity Theta Burst Stimulation. Clin. Neurophysiol..

[31] Chung SW, Hill AT, Rogasch NC, Hoy KE, Fitzgerald PB (2016). Use of theta-burst stimulation in changing excitability of motor cortex: A systematic review and meta-analysis. Neurosci. Biobehav. Rev..

[32] Chung SW, Rogasch NC, Hoy KE, Fitzgerald PB (2018). The effect of single and repeated prefrontal intermittent theta burst stimulation on cortical reactivity and working memory. Brain Stimul.

[33] Gentner R, Wankerl K, Reinsberger C, Zeller D, Classen J (2008). Depression of human corticospinal excitability induced by magnetic theta-burst stimulation: evidence of rapid polarity-reversing metaplasticity. Cereb. Cortex.

[34] Iezzi E (2008). Phasic voluntary movements reverse the aftereffects of subsequent theta-burst stimulation in humans. J. Neurophysiol..

[35] Goldsworthy MR, Müller-Dahlhaus F, Ridding MC, Ziemann U (2014). Inter-subject variability of LTD-like plasticity in human motor cortex: a matter of preceding motor activation. Brain Stimul..

[36] Doeltgen SH, Ridding MC (2011). Low-intensity, short-interval theta burst stimulation modulates excitatory but not inhibitory motor networks. Clin. Neurophysiol..

[37] Huang YY, Colino A, Selig DK, Malenka RC (1992). The influence of prior synaptic activity on the induction of long-term potentiation. Science.

[38] Christie BR, Abraham WC (1992). Priming of associative long-term depression in the dentate gyrus by theta frequency synaptic activity. Neuron.

[39] Kramár EA (2012). Synaptic evidence for the efficacy of spaced learning. Proc. Natl. Acad. Sci. USA.

[40] Volz LJ, Benali A, Mix A, Neubacher U, Funke K (2013). Dose-dependence of changes in cortical protein expression induced with repeated transcranial magnetic theta-burst stimulation in the rat. Brain Stimul..

[41] Goldsworthy MR, Pitcher JB, Ridding MC (2013). Neuroplastic modulation of inhibitory motor cortical networks by spaced theta burst stimulation protocols. Brain Stimul..

[42] Zafar N, Paulus W, Sommer M (2008). Comparative assessment of best conventional with best theta burst repetitive transcranial magnetic stimulation protocols on human motor cortex excitability. Clin. Neurophysiol..

[43] Rossi S, Hallett M, Rossini PM, Pascual-Leone A, Safety of TMS Consensus Group (2009). Safety, ethical considerations, and application guidelines for the use of transcranial magnetic stimulation in clinical practice and research. Clin. Neurophysiol..

[44] Peurala SH, Müller-Dahlhaus JFM, Arai N, Ziemann U (2008). Interference of short-interval intracortical inhibition (SICI) and short-interval intracortical facilitation (SICF). Clin. Neurophysiol..

[45] Ridding MC, Taylor JL, Rothwell JC (1995). The effect of voluntary contraction on cortico-cortical inhibition in human motor cortex. J. Physiol..

